# T Cells Specifically Targeted to Amyloid Plaques Enhance Plaque Clearance in a Mouse Model of Alzheimer's Disease

**DOI:** 10.1371/journal.pone.0010830

**Published:** 2010-05-26

**Authors:** Yair Fisher, Anna Nemirovsky, Rona Baron, Alon Monsonego

**Affiliations:** The Shraga Segal Department of Microbiology and Immunology, Faculty of Health Sciences, and The National Institute for Biotechnology in the Negev, Ben-Gurion University of the Negev, Beer-Sheva, Israel; Massachusetts General Hospital and Harvard Medical School, United States of America

## Abstract

Patients with Alzheimer's disease (AD) exhibit substantial accumulation of amyloid-β (Aβ) plaques in the brain. Here, we examine whether Aβ vaccination can facilitate the migration of T lymphocytes to specifically target Aβ plaques and consequently enhance their removal. Using a new mouse model of AD, we show that immunization with Aβ, but not with the encephalitogenic proteolipid protein (PLP), results in the accumulation of T cells at Aβ plaques in the brain. Although both Aβ-reactive and PLP-reactive T cells have a similar phenotype of Th1 cells secreting primarily IFN-γ, the encephalitogenic T cells penetrated the spinal cord and caused experimental autoimmune encephalomyelitis (EAE), whereas Aβ T cells accumulated primarily at Aβ plaques in the brain but not the spinal cord and induced almost complete clearance of Aβ. Furthermore, while a single vaccination with Aβ resulted in upregulation of the phagocytic markers triggering receptors expressed on myeloid cells-2 (TREM2) and signal regulatory protein-β1 (SIRPβ1) in the brain, it caused downregulation of the proinflammatory cytokines TNF-α and IL-6. We thus suggest that Aβ deposits in the hippocampus area prioritize the targeting of Aβ-reactive but not PLP-reactive T cells upon vaccination. The stimulation of Aβ-reactive T cells at sites of Aβ plaques resulted in IFN-γ-induced chemotaxis of leukocytes and therapeutic clearance of Aβ.

## Introduction

Alzheimer's disease (AD), an age-related neurodegenerative disorder, is the most common cause of dementia. The disease is identifiable by the accumulation of amyloid beta (Aβ) in the hippocampal and cortical areas of the brain [1], often associated with neurofibrillary tangles caused by hyperphosphorylation of the cytoskeleton protein Tau [2]. Aβ appears to exert a toxic effect on neurons—both directly in its oligomeric form [3] and indirectly in the form of dense plaques—by inducing chronic activation of microglia and astrocytes [4].

The role of T cells in central nervous system (CNS) tissue has been widely studied in recent years. Findings have primarily implicated CNS-specific CD4 T cells in the pathology of experimental autoimmune encephalomyelitis (EAE), where myelin-specific T cells penetrate the CNS and promote axon demyelination there [5], [6], [7], [8], [9]. Despite the pathogenic role of T cells in mouse models of multiple sclerosis (MS), brain-specific T cells evidently play beneficial roles in non-autoimmune neurodegenerative processes, such as those occurring in Alzheimer-like disease [10], [11], brain injury [12], [13], amyotrophic lateral sclerosis [14] and stroke [15]. Activities in which these specific T cells, or the cytokines they produce, were found to participate include increased uptake of cell debris by microglia [16], release of anti-inflammatory cytokines [17], increased expression of neurotrophic factors [11], [18], [19], increased capacity for buffering of glutamate toxicity[20], and enhanced neurogenesis [21], [22], [23], [24]. We recently demonstrated the presence of Aβ-specific T cells in the repertoires of human subjects, depending primarily on their HLA–DR genetic backgrounds [25], [26]. We showed that the frequency of these T cells is significantly increased both in elderly healthy individuals and in patients with AD [25]. We also characterized the specificities of such T cells in humanized mouse AD models expressing frequent HLA–DR alleles [26].

Interferon (IFN)-γ is a natural-killer (NK)-cell and T-cell-derived protein that plays a key role in the effector function of the immune system against invading pathogens, activating microglia to fully potent antigen-presenting cells [27] and enhancing the expression of chemokines and cell-adhesion molecules required for migration of T-cells to the brain [28]. However, beyond its primary role in inflammation, recent findings demonstrate that IFN-γ expression is increased in the ageing brain [29] and exerts neuroprotective effects [22], [30], [31], [32], [33], [34]. To gain an insight into the mechanisms by which Aβ-specific T cells function at sites of Aβ plaques, we generated a mouse model of AD in which the brain expresses IFN-γ in small amounts that do not cause spontaneous infiltration of bone marrow-derived cells, abnormal glial activation, or neurological deficits [35]. A single immunization of such mice with Aβ, but not with the encephalitogenic proteolipid protein (PLP 139-151), resulted in trafficking to the brain of immune cells, which locally altered the cytokine milieu and enhanced the clearance of Aβ.

## Materials and Methods

### Mice

C57BL6 and SJL mice were purchased from Harlan Israel. APP-Tg mice (line J20) on a C57BL6 background expressing APP under the platelet-derived growth factor promoter [36] were kindly donated by Dr. Mucke. APP/PS1-Tg mice [37] were kindly supplied by Dr. Mathias Jucker. Transgenic SJL mice expressing IFN-γ under the myelin basic protein promoter [35] were kindly donated by Dr. Owens. Homozygous IFN-γ-Tg mice were crossed with either APP-Tg or APP/PS1-Tg mice to generate APP/IFN-γ or APP/PS1/IFN-γ B6SJLF1 Tg mice, respectively. All surgical and experimental procedures were reviewed and approved by the Institutional Animal Care and Use Committee (IACUC) of Ben-Gurion University of the Negev (Approval number IL-610904).

### Immunization

Mice (9–10 months old) were immunized by injection into the flank with Aβ_1-42_ (100 µg) or proteolipid protein (PLP)_139-151_ (400 µg; GenScript Corp., Piscataway, NJ, USA) emulsified in complete Freund's adjuvant (CFA) H37Ra (Difco, Detroit, MI, USA). The Aβ_1-42_ peptide used for immunization was initially dissolved in a small volume of DMSO to enhance its solubility and then diluted in phosphate buffer saline (PBS) to 2 mg/ml. The peptide existed primarily in a trimer form as indicated by gradient gel (4–20%) electrophoresis and western blot analysis using the 6E10 anti-Aβ monoclonal antibodies (data not shown) and as such was emulsified with CFA to a final concentration of 1 mg/ml. Pertussis toxin (PTX) (List Biological Laboratories, Campbell, CA, USA) was injected i.v. (200 ng/mouse) on day 0 and again 48 h later. EAE severity was scored as follows: grade 1, paralyzed tail; grade 2, ataxic; grade 2.5, one hind leg paralyzed; grade 3, both hind legs paralyzed; grade 3.5, three legs paralyzed; grade 4, complete paralysis; grade 5, moribund.

### Cytokine ELISA

Popliteal lymph nodes or spleens of immunized APP/IFN-γ Tg mice were harvested, and their cells (10×10^6^ cells/ml) were cultured in U-shaped 96-well-plate culture dishes in DMEM medium containing 10% fetal calf serum, 10 mM HEPES, 1 mM sodium pyruvate, 10 mM nonessential amino acids, 1% Pen/Strep and 50 µM β mercaptoethanol. Interleukin (IL)-2 and IL-4 were measured in the supernatant after 24 h, IFN-γ and IL-10 after 48 h, and IL-17A after 72 h, in each case with a sandwich ELISA according to the manufacturer's instructions (Biolegend, San Diego, CA, USA).

### Quantitative real-time PCR

Mice were anesthetized by i.p. injection of ketamine-xylazine and perfused with ice-cold PBS buffer. The brain was removed from the skull, and the hippocampus-cortex was excised on ice. Total RNA was extracted with Bio-Tri RNA (Bio-Lab, Jerusalem, Israel) according to the manufacturer's instructions. Total RNA (5 µg) was reverse^-^transcribed using a Bio-RT kit (Bio-Lab, Jerusalem, Israel) according to the manufacturer's instructions. The primers for real time (RT)-PCR were designed using Primer Express software (Applied Biosystems, Warrington, UK) and synthesized by Sigma. Real-time quantification of genes was performed by means of a SYBER Green RT-PCR assay (Applied Biosystems) using 125 ng of total cDNA for 20 µl of the RT-PCR reaction mixture. Samples were run in triplicate, and the expression level of each gene was compared with that of β-actin. Amplification, detection of specific gene products, and quantitative analyses were performed using the ABI PRISM® 7500 Sequence Detection System (Applied Biosystems).

### Immunohistochemistry

Mice were killed with an overdose of isoflurane, and their brains were rapidly removed and placed in molds containing an OCT compound (Tissue-Tek, Torrance, CA, USA). The tissues were frozen in isopentane, cooled by liquid nitrogen, and stored at −80°C. Sagittal sections (12 µm) were taken throughout the hippocampus and fixed in ice-cold methanol for 2 min, then in 4% formaldehyde for 4 min, and then washed with DDW and PBS/Tween (0.05%). Prior to staining, primary antibody diluting buffer (Biomeda Corp., Foster City, CA, USA) was used to block nonspecific binding. An avidin-biotin blocking kit (Vector Labs, Burlingame, CA, USA) was used when sections were stained with biotinylated primary antibodies. Sections were examined under an Olympus Fluoview FV1000 laser scanning confocal microscope.

#### Axonal demyelination

For detection of myelin loss, frozen brain sections were stained with Luxol Fast Blue solution (1% Luxol fast blue, Sigma-Aldrich; 10% acetic acid, 95% ethanol) at 60°C for 2 h. Slides were rinsed in 95% ethanol and then in DDW. They were then incubated in a 0.05% lithium carbonate solution (Fluka Analytical) in DDW for 10 s, and then for 30 s in 70% ethanol followed by DDW for 30 s. These differentiating steps were repeated until grey and white matter were clearly distinguishable. Slides were then counterstained with 0.25% cresyl echt violet solution (Fluka Analytical; 0.25% cresyl echt violet, 10% acetic acid in DDW) for 10 min at room temperature, and washed in DDW and then in 70% and 100% ethanol. Finally, slides were cleared and mounted with VectaMount (Vector Labs).

### Antibodies

PECAM-1 (1∶50), CD4 (1∶50), CD8a (1∶50), CD19 (1∶50), and CD11c (1∶25) were all purchased from Biolegend, San Diego, CA, USA. CD11b (1∶25) was obtained from Serotec, Raleigh, NC, USA. Rabbit anti-human Aβ antibodies (1∶500) were generated at our animal facility and examined for specificity by ELISA and immunohistochemistry. TO-PRO 3 (Invitrogen, Carlsbad, CA, USA) was used in a 1∶3000 dilution, and all subsequent secondary antibodies or avidin were conjugated to Alexa-488, Alexa-546, or Alexa-647 (Molecular Probes) diluted 1∶500 and 1∶800 for antibodies and avidin, respectively.

### Confocal imaging analysis

#### CD4/CD8 co-localization in the hippocampus

Brain sections from immunized APP/IFN-γ Tg mice were immunostained with anti-CD4 and anti-CD8 for the analysis of CD4 and CD8 T cells, respectively, and with PECAM-1 and Aβ to locate lymphocytes in the vasculature and Aβ plaques, respectively. Areas of the hippocampus, including the dentate gyrus, were imaged with both the 10× and 60× objective lenses, and T-cell subpopulations were counted manually.

#### Quantification of Aβ plaques

APP/IFN-γ Tg mice were immunized with Aβ, with or without injection of PTX. Brains from untreated and treated mice were quantitatively analyzed for Aβ plaques using three–six sections (40 µm thick) per hemisphere for accurate representation of the hippocampus area. Sections were stained for Aβand examined with a confocal microscope. Fluorescence intensity was first obtained in sections from untreated mice, and identical laser-scanning parameters were then used for the entire experiment. Using Volocity 3D image analysis software (Improvision, Waltham, MA, USA), we set an intensity threshold to mark only those areas stained with Aβ (Supplementary data, Fig. S1). The sum of the stained areas was calculated in three regions of the hippocampus: 0.5–0.9 mm, 0.9–1.35 mm, and 1.35–1.8 mm mediolateral from the bregma (Mouse Brain Atlas, Franklin and Paxinos 2007). The average of the summed fluorescence area was calculated for each group per hippocampus location (2–4 slices/location).

## Results

### Immunization of APP/IFN-γ Tg mice with Aβ, but not with PLP, results in the accumulation of T cells primarily at sites of Aβ plaques

**Figure 1:**
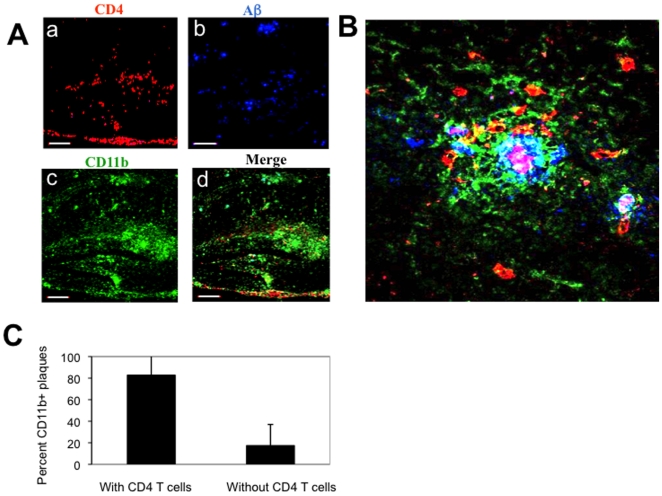
Aβ immunization results in trafficking of T cells to sites of Aβ plaques in the brain parenchyma. APP/IFN-γ Tg mice aged 9 months were immunized with Aβ and killed 19 days later. Brain sections were immunolabeled for Aβ plaques co-localized with lymphocyte subpopulations and activated microglia. (**A**) Brain sections derived from a representative Aβ-immunized APP/IFN-γ Tg mouse (*n* = 11) were immunolabeled with anti-CD4 (*a*, red), anti-Aβ (*b*, blue), and anti-CD11b (*c*, green) antibodies. The merged panel is shown in *d*. Bar represents 200 µm. (**B**) Three-dimensional representation of Z-stalk images taken from a representative compact Aβ plaque co-localized with CD11b^+^ microglia (green) and CD4 T cells (red) in the hippocampus. Bar represents 20 µm. (**C**) Co-localization analysis of CD4 T cells and CD11b-labeled plaques. Using Volocity 3D image analysis software (Improvision, Waltham, MA, USA), we set an intensity threshold to mark only those areas with CD11b^high^ microglia, representing Aβ plaques. Columns represent percent CD11b-labeled plaques with and without co-localized CD4 T cells in each of the analyzed sections [*n* = 3 (3 brain sections per mouse); means ± SD; *P*<0.001, Student's *t* test].

A previous study by our group demonstrated that small amounts of IFN-γ in the brain allowed lymphocyte migration to the brains of APP-Tg mice upon eliciting Aβ-specific Th1 type of immune response [16]. Aβ immunization of APP-Tg mice lacking IFN-γ in the brain, although elicited the same peripheral immune response, did not result in T-cell occurrence at both the meningeal and parenchymal tissues of the brain [16]. However, although APP is expressed throughout the CNS, we observed certain differences in Aβ-immunized APP/IFN-γ Tg mice with regard to the pattern of T cells in the CNS compared to the pattern usually observed in mice that develop EAE upon immunization with myelin peptides. First, the target site of migration in the CNS was found to be the brain but not the spinal cord [16]. Second, lymphocytes appeared not to generate chronic lesions around the vessels but rather to migrate further into the parenchyma. To test our hypothesis that immunization of APP/IFN-γ Tg mice with Aβ, but not with an encephalitogenic peptide, results in lymphocyte migration primarily to sites of Aβ plaques in the brain, we immunized mice with Aβor the encephalitogenic PLP peptide 139–151, and 19 days later we co-stained brain sections for Aβ, microglia and T cells. We found that CD4 T cells (Fig. 1Aa and Supplemental data, Fig. S2a-b) were co-localized with Aβ plaques (Fig. 1Ab and Supplemental data, Fig. S2c-d) and CD11b^+^ microglia (Fig. 1Ac and Supplemental data, Fig. S2e-f) primarily in areas of the hippocampus, including the dentate gyrus, where accumulation of Aβ in this mouse model of the disease is more intense in the initial deposition process [36]. A three-dimensional reconstructed image demonstrated that lymphocytes at the core of the Aβ plaque deposition were co-localized with CD11b microglia (Fig. 1B). Co-localization image analysis of CD4 T cells and Aβ plaques demonstrated that T cells in the brain were specifically targeted to sites of Aβ plaques (Fig. 1C).

**Figure 2:**
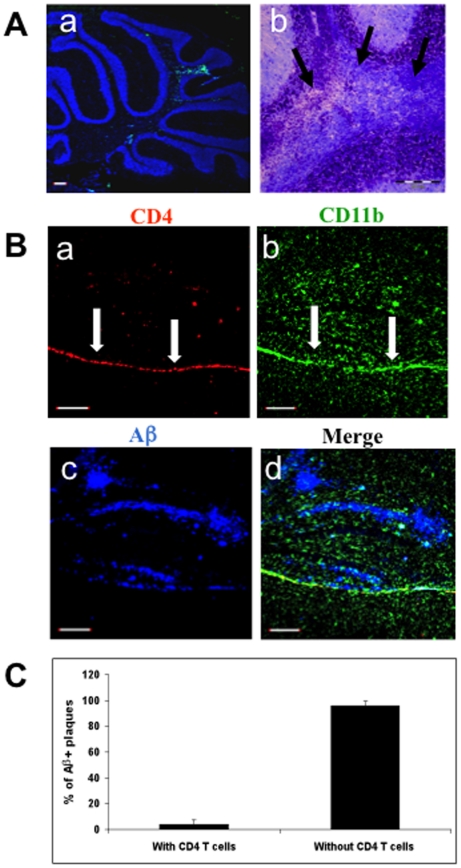
PLP immunization results in limited T-cell occurrence at the hippocampus of APP Tg mice. APP/IFN-γ Tg mice aged 9 months (*n* = 5) were immunized with PLP and killed 19 days later. (**A**) Brain sections were analyzed for leukocyte infiltrates as described in Materials and Methods. Sections were counterstained with TO-PRO 3 (blue). (**a**) Brain section showing infiltrating CD4 T cells (green) in the cerebellum. (**b**) Magnified area of the cerebellum stained with Luxol Fast Blue, demonstrating areas with infiltrated cells and loss of myelin (black arrows). Bar represents 200 µm. **(B)** Brain sections derived from a representative PLP-immunized APP/IFN-γ Tg mouse were immunolabeled with anti-CD4 (**a**, red), anti-CD11b (**b**, green), and anti-Aβ (**c**, blue) antibodies. The merged panel is shown in **d**. Bar represents 200 µm. (**C**) Co-localization analysis of CD4 T cells and Aβ-labeled plaques. Using Volocity 3D image analysis software (Improvision, Waltham, MA, USA), we set an intensity threshold to mark only those areas with Aβ plaques. Columns represent percent Aβplaques with and without co-localized CD4 T cells in each of the analyzed sections [*n* = 3 (3 brain sections per mouse); means ± SD].

**Figure 3:**
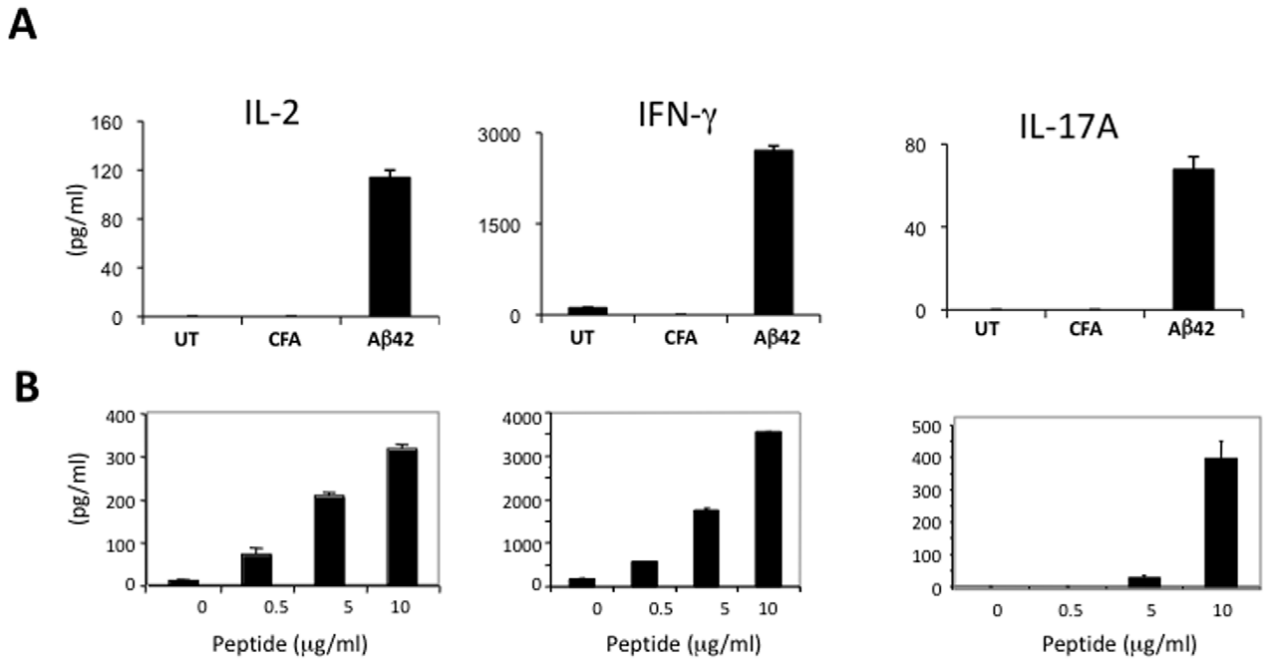
Aβ or PLP vaccination of APP-Tg mice induces primarily a Th1 type of immune response. APP/IFN-γ Tg mice aged 9–10 months were injected subcutaneously with Aβ (**A**) or PLP (**B**) emulsified in CFA in conjunction with PTX injections. Control mice were injected with CFA or left untreated (**A**). Mice were killed 10 days later, and popliteal lymph nodes were harvested and analyzed for cytokine secretion by ELISA as described in Materials and Methods. Lymph node derived cells were cultured without antigen or stimulated with increasing concentrations of Aβ and PLP (0.1, 1 and 10 µg/ml). Secretion of IL-2, IFN-γ, and IL-17A was measured after 24, 48, and 72 h in culture, respectively. Data obtained at 10 µg/ml Aβ are shown in A. The results shown in each case are the values obtained for three mice (means ± SD) in one representative experiment of at least three performed.

In contrast to immunization with Aβ, immunization of APP/IFN-γ Tg mice with PLP induced EAE with a clinical score of 2–3 (see EAE scoring scheme in Materials and Methods). In addition, PLP immunization resulted in the appearance of CD4 T-cell infiltrates in the spinal cord (Supplemental data, Fig. S3) and cerebellum (Fig. 2Aa) and typical demyelinating lesions, as indicated by Luxol Fast Blue staining (Fig. 2Ab, see arrows). Moreover, although CD4+ (Fig. 2Ba and Supplemental data, Fig. S4a-b) and CD11b+ (Fig. 2Bb and Supplemental data, Fig. S4e-f) cells accumulated at the meninges following to PLP immunization, only a few T cells were found in the hippocampus co-localized with Aβ deposits (Fig. 2B–C and Supplemental data, Fig. S4c-d). To determine whether this difference in the migration pattern of Aβ- reactive and PLP- reactive T cells to the CNS of APP-Tg mice resulted from the cytokine profile of the cells, mice were immunized with Aβor PLP and T cells were examined *ex vivo* for cytokine secretion by ELISA. Notably, a similar Th1-cell response was induced both after PLP and after Aβ immunization (Fig. 3). Upon antigen stimulation, T cells secreted primarily IL-2 as the growth factor cytokine and IFN-γ and—though to a substantially smaller extent—IL-17A as the effector cytokines (Fig. 3A-B). No significant cytokine production was observed in untreated or CFA-immunized mice upon Aβstimulation (Fig. 3A).

### Aβ immunization of APP/IFN-γ Tg mice results in migration primarily of CD4 and CD8 T cells to the brain parenchyma

**Figure 4:**
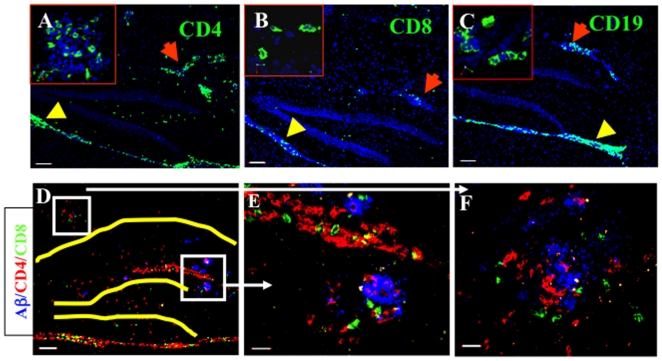
Aβ immunization results in immune-cell trafficking to the brain. APP/IFN-γ. Tg mice aged 9 months were immunized with Aβ and killed 19 days later, and brain sections were immunolabeled as described in Materials and Methods. Lymphocyte subpopulations immunostained for CD4 (**A**, green), CD8 (**B**, green), and CD19 (**C**, green, with red arrows indicating infiltrates in parenchymal vessels). Yellow arrowheads indicate meningeal infiltrates. Sections were counterstained with TO-PRO 3 (blue). Bars represent 100 µm; inserts were taken with a 60× objective lens. (**D**) Overview of the hippocampal area (hippocampus and dentate gyrus indicated in yellow) showing CD4 (red) and CD8 (green) T cells co-localized with Aβ plaques (blue). Bar represents 100 µm. Higher magnification of Aβ plaques (blue) co-localized with CD4 (red) and CD8 (green) T cells adjacent to a parenchymal blood vessel in the dentate gyrus (**E**) and the CA1 (**F**) of the hippocampal formation. Bars represent 20 µm. Representative images from eight mice are shown.

To further characterize the location and type(s) of cells infiltrating into the hippocampal and meningeal tissues of the brain, APP/IFN-γ Tg mice were killed 19 days after immunization, and their brains were excised and processed for immunohistochemistry. Both CD4 T cells (Fig. 4A) and CD8 T cells (Fig. 4B) were observed in the meningeal (yellow arrowheads) and parenchymal blood vasculature (red arrows). CD19 B cells were found in the meninges (yellow arrowheads) and parenchymal vessels (red arrows), but not in the parenchymal tissue (Fig. 4C). No natural killer cells were observed anywhere in the brain at 14 to 19 days post-immunization (data not shown). In addition, co-staining of Aβ (blue), CD4 (red), and CD8 (green) T cells revealed the occurrence of both of these lymphocyte subpopulations in the perivascular space (Fig. 4D–E) and at amyloid plaques (Fig. 4E–F). Quantification of CD4 and CD8 T cells in the brains of the Aβ-immunized mice revealed that CD4 effector T cells comprised the dominant population (64.3%, whereas CD8 cells comprised 35.7%; *n* = 3, SD = 7.74). These data suggest that in Aβ-immunized mice these two T-cell subtypes not only cross the blood–brain barrier but also home to amyloid plaques, where they appear to be activated, presumably as a result of restimulation by local antigen-presenting cells (APCs).

### Aβ immunization of APP/IFN-γ Tg mice results in effective clearance of Aβ plaques co-localized with T cells

**Figure 5:**
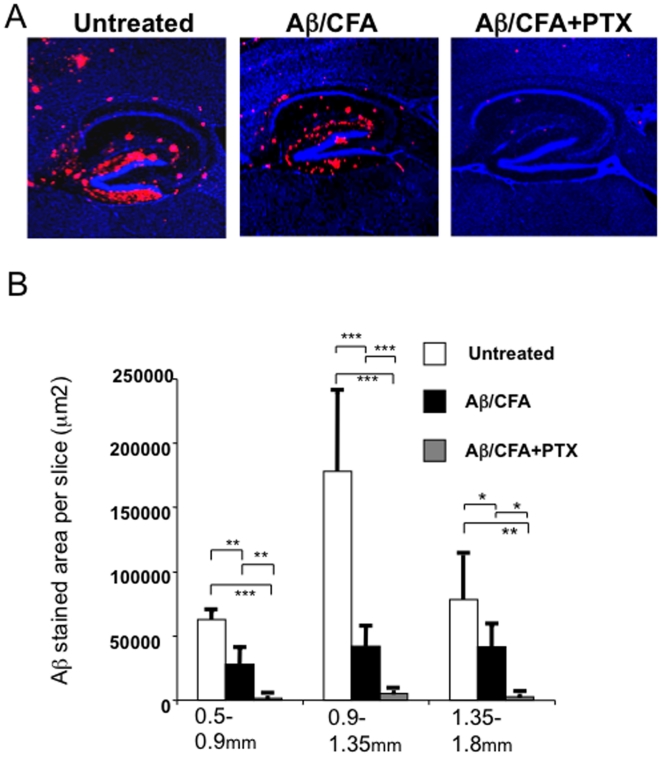
T-cell infiltrates in the parenchyma promote efficient clearance of existing Aβ plaques. APP/IFN-γ Tg mice aged 9 months were immunized with Aβ emulsified in CFA, Aβ emulsified in CFA in conjunction with injection of PTX, or left untreated and examined 5 weeks later for Aβ in the brain. (**A**) Brain sections were immunolabeled with anti-Aβ antibody (red) and counterstained with TO-PRO 3 (blue). A representative section from each group is shown. (**B**) The amount of Aβ in each group was quantified stereologically on 40-mm-thick sections using the Volocity 3S Image Analysis software, as described in Materials and Methods and in Supplemental data, Fig. S4. One-tailed Student's *t*-test revealed significant differences between the three groups of mice (*n* = 4 mice/group; *P<0.05, **P<0.01, ***P<0.001).

Post-mortem examination of AD patients vaccinated with Aβ has revealed clear evidence of plaque removal up to five years after immunization [38], [39]. To determine whether the activated T cells residing at sites of Aβ plaques could enhance the clearance of Aβ from the brain, we immunized 9-month-old APP/IFN-γ Tg mice with a single injection of Aβ_1-42_ emulsified in CFA with or without PTX. Mice were killed five weeks later, and their brains were processed for immunohistochemistry and quantification of Aβ, as described in Materials and Methods. T cells were visible only in the brains of mice that had been immunized with Aβ/CFA and co-injected with PTX, and the T-cell pattern observed in those brains (not shown here) was similar to that shown in Fig. 1. As shown in Fig. 5A, Aβ/CFA immunization without PTX acted primarily to reduce diffuse forms of Aβ plaques, whereas Aβ/CFA immunization with co-injected PTX was followed by almost complete clearance of Aβ from the brain. In three different regions mediolateral to the bregma, stereological quantification of Aβ showed significantly reduced average amounts of Aβ both without (60% reduction) and with (97% reduction) PTX co-injection (Fig. 5B, and Supplemental data, Fig. S1 and Table S1). No significant clearance of Aβ was observed in mice immunized with PLP (Supplemental data, Fig. S5) or CFA alone (Supplemental data, Fig. S6).

To gain an insight into the inflammatory milieu induced in the brains of APP/IFN-γ Tg mice after T cells had migrated to sites of Aβ plaques, we performed quantitative PCR analysis of brain tissues from mice that had been immunized with Aβ/CFA and co-injected with PTX. Nonimmunized APP/IFN-γ Tg mice served as controls. Consistent with the Th1 phenotype of the peripheral T-cell response and the accumulation of T cells observed in the brain, IFN-γ was substantially increased in the brain (by 39.7-fold), as was the case for the T-cell and macrophage chemoattractant RANTES (by 39.8-fold) and the IFN-γ-inducible protein IP-10 (by 20.3-fold; Table 1). Expression of the MHC II invariant chain and of the costimulatory molecule CD86 (Table 1) was also enhanced, suggesting that T cells had not only migrated to the brain but had also undergone activation at sites of Aβ plaques, as indicated by their morphology. Notably, although there was no increase in the anti-inflammatory cytokines IL-10 and transforming growth factor (TGF)- β, the proinflammatory cytokines IL-6 and TNF-α were decreased (by about 3.3-fold) in the immunized mice (Table 1). Moreover, the signal regulatory protein-β1 (SIRPβ1) and the triggering receptor expressed on myeloid cells-2 (TREM2) – transmembrane molecules recently shown to be associated with enhanced phagocytic activity of macrophages/microglia [40], [41] – were upregulated by 7.3-fold and 2.8-fold, respectively (Table 1).

**Table 1:** Quantitative PCR analysis of immune factors in the brains of Aβ-immunized APP/IFN-γ Tg mice.

Gene	RQ	*P* value
**Antigen presentation**		
Invariant chain	12.72±5.7	0.0148
CD86	5.5±3.3	0.0223
**Immune regulators**		
INF-γ	39.7±18.9	0.0013
IL-6	0.3±0.12	0.0278
TNF-a	0.29±0.05	0.0278
IL-10	undetected	
TGF-β	no difference	
**Chemokines**		
RANTES	39.8±23.3	0.0075
IP-10	20.3±8.5	0.0020
**Microglial activation**		
SIRP1β	7.3±0.15	0.0002
TREM2	2.8±0.03	0.0010

APP/IFN-γ Tg mice aged 9 months were immunized with Aβ/CFA and co-injected twice with PTX as described in Methods. Untreated APP/IFN-γ Tg mice were used as controls. Mice were analyzed for gene expression in the brain by quantitative PCR as described in Materials and Methods. For each of the genes analyzed, the relative quantity (RQ) was calculated in relation to the level of actin expression. Results are presented as average RQs of the expressed genes in vaccinated mice relative to control mice. *P* values and SEM were calculated from two immunized mice with substantial T-cell infiltrates in the brain parenchyma and four untreated mice, using the 2-tailed Mann-Whitney test.

## Discussion

The results of this study show that in a mouse model of AD, Aβ-specific lymphocytes occurred in the brain after a single Aβ immunization, provided that small amounts of IFN-γ were expressed in the brain. Although both T and B lymphocytes crossed the vasculature into the perivascular space, only the T cells were targeted to Aβ plaques in the brain parenchymal tissue, where they induced microglia activation and efficient clearance of Aβ. In contrast, PLP immunization resulted in T-cell accumulation in the spinal cord and cerebellum, causing demyelination, but not in the hippocampus, where Aβ accumulated.

An important question that stems from the current study is why Aβ-reactive but not PLP-reactive T cells preferentially target Aβ plaques in the hippocampus. Several studies have previously demonstrated the presence of perivascular cells capable of presenting antigens to T cells in the endothelium of the brain microvessels [7], [42], [43], [44]. It is possible that these cells ensure that, in the absence of an overwhelming inflammatory response, only brain-specific T cells enter the CNS. In such a case, the APCs – by engulfing antigens in the brain vasculature – could facilitate both the stimulation of specific CD8 and CD4 T cells (via MHC class I and II, respectively) and the trafficking of these T cells to the parenchyma. Aβ is deposited in the vasculature throughout the course of AD [45]. Furthermore, post-mortem examination of brain sections of AD patients who had been immunized with Aβ showed a transient increase in the severity of cerebral amyloid angiopathy, presumably as a consequence of enhanced drainage of Aβ into the vasculature [38]. Expression of TGF-β in the brain of a mouse model of AD was also shown to result in the accumulation of Aβ in the cerebral blood vessels [46] and spontaneous infiltration of T cells into the brain parenchyma, a process that was significantly enhanced upon long-term Aβ immunization [47]. It therefore seems likely that drainage of Aβ into the perivascular space and its local deposition there promote activation of Aβ-specific T cells, thereby enhancing the ability of these T cells, but not of PLP-reactive T cells, to specifically target Aβ plaques in the parenchyma.

The T-cell responses observed in the current study in the brain of a mouse model of AD differed in two important aspects from those of EAE, in which myelin-specific T cells penetrate the CNS and promote the demyelination of axons [5], [6], [8], [48]. Firstly, the T-cell response that we observed in the CNS did not lead to a chronic influx of CD4 T cells into the CNS of our mouse model. This finding might be explained by the fact that Aβ deposits were efficiently cleared from the brain and hence the lymphocyte response was terminated. Secondly, our study clearly demonstrated that whereas PLP-specific T cells caused EAE lesions in the spinal cord, Aβ-specific T cells acted primarily to target amyloid deposits in the brain: Aβ-specific T cells in our study did not enter the brain if there was no amyloid deposition there [16]. These data suggest that unlike mouse models of MS where T cells penetrate the intact CNS, Aβ accumulation and deposition in the brain is a precondition for triggering the entry of specific T cells upon immunization.

The fact that Aβ accumulates primarily in the hippocampus and the frontal cortex also provides the Aβ-specific T cells with a parenchymal target for migration and activation. As shown in Fig. 4, both CD4 T cells and, to a significantly smaller extent, CD8 T cells were targeted to sites of Aβ plaques in the hippocampus. CD4 T cells migrated towards sites of Aβ accumulation and were presumably restimulated there by microglia via MHC-II presentation of Aβ, with consequent activation of their effector function within the parenchymal tissue (Table 1). Because the mice in this study were immunized with Aβ_1-42_, it is possible that Aβ-specific CD8 T cells in the peripheral lymph nodes were also stimulated and could thus migrate to the CNS via either endothelial or dendritic cells presenting Aβ on MHC-I. It is also possible that CD8 T cells that migrate into the parenchyma interact with both glia and neurons presenting Aβ on MHC I. The specificity and function of these cells in the brain of a mouse model of AD should therefore be further investigated.

The role of T cells in the CNS tissue has been widely studied in recent years. A growing body of evidence has indicated that T-cell trafficking to the brains of APP-Tg mice overexpressing TGF-β or IL-1β does not cause cellular or behavioral abnormalities [47], [49] and that brain-specific T cells can exhibit beneficial effects by regulating the neuroinflammatory environment and the mechanisms of neuronal protection and repair in several neurodegenerative processes [10], [11], [12], [13], [15], [16], [18], [50]. In the present study, Aβ-specific T cells displayed a Th1 phenotype in that they secreted primarily IFN-γ (Table 1), a key cytokine required for stimulating the phagocytotic and degradative activities of macrophages. Notably, both TREM2 and SIRPβ1, recently shown as DAP12-associated phagocytic receptors on microglia [40], [41], were upregulated by 2.8-fold and 7.3-fold, respectively (Table 1). Furthermore, our quantitative PCR analysis showed that this increased expression of TREM2 and SIRPβ1 was associated with decreased expression of TNFβ and IL-6, suggesting that the activation of microglia enhanced their phagocytic function but the overall inflammatory process. Indeed, Gaikwad et al. [41] have recently demonstrated that IFN-γ induces the expression of SIRPβ1 in microglia and that activation of SIRPβ1 by specific antibodies enhances the uptake of fibrillar Aβ while suppressing lipopolysaccharide (LPS)-induced TNFα secretion. Activation of Th1 cells in the brain, therefore, presumably accounted for the activation of microglia and the efficient clearance of Aβ, as was previously achieved by prophylactic periodic immunizations that evoked high titers of Aβ antibodies [51], [52], [53], [54], [55], [56]. In light of the regulatory and neuroprotective functions of IFN-γ in the brain [20], [22], [32], [33], [34], [57], [58], future studies should therefore address the effects of such IFN-γ-secreting T cells at sites of Aβ plaques on microglia function, the immune milieu induced locally, and the consequent survival and function of adjacent neurons.

Even though it did not induce EAE, the lymphocytic reaction of both B and T cells to Aβ is potentially pathogenic because of the risks of meningoencephalitis [59], entry of cytotoxic CD8 T cells into the brain, strong proinflammatory cytokine profile of the CD4-T cells [60], and brain hemorrhages caused by Aβ antibodies [61], [62], [63]. However, studies by our group have demonstrated a remarkably increased frequency of Aβ-specific T cells in healthy older individuals and in patients with AD [25]. If the pathogenic capacity of Aβ-specific T cells can be neutralized, for example, by using a bacteria-free or virus-free adjuvant such as alum or incomplete Freund's adjuvant, these T cells might play a clinical role in promoting both Aβ clearance and neuronal repair [19], with minimal risk of adverse side effects.

## Supporting Information

Figure S1 T-cell infiltrates in the parenchyma promote efficient clearance of previously deposited Aβ plaques. APP/IFN-γ Tg mice aged 9 months were immunized with Aβ emulsified in CFA, Aβ emulsified in CFA in conjunction with PTX injections, or left untreated, and examined 35 days later for Aβ in the brain. Brain sections were immunolabeled with anti-Aβ antibody (red) and counterstained with TO-PRO 3 (blue), as described in Materials and Methods. A representative section from each group is shown in A. Higher magnification (×40) of the box in A is shown for each group in panel B. Plaques are marked with colored lines if their fluorescence intensity exceeds the threshold set by the classifier.
(3.00 MB TIF)

Figure S2 Aβ immunization results in trafficking of T cells to sites of Aβ plaques in the brain parenchyma. APP/IFN-γ Tg mice aged 9 months were immunized with Aβ and killed 19 days later. Brain sections were immunolabeled for Aβ plaques co-localized with lymphocyte subpopulations and activated microglia as described for Fig. 1. The grayscale images with their corresponding histograms are shown here at 10× (a, c, and e; bars represent 200 µm) and 60× (b, d, and f; bars represent 20 µm).
(3.00 MB TIF)

Figure S3 PLP vaccination of APP/IFN-γ Tg mice results in immune-cell infiltration into the spinal cord and cerebellum. APP/IFN-γ Tg mice aged 9 months were vaccinated with PLP/CFA to induce EAE, as described in Materials and Methods, and killed 19 days later. Spinal cord sections were immunolabeled with anti-CD11b antibody (a and b, green), anti-CD11c antibody (c and d, red), and anti-CD4 antibody (red in a and b and green in c and d) and examined under the confocal microscope for inflammatory foci. TO-PRO 3 was used for counterstaining (blue). (b and d) Higher magnifications of inflammatory foci. Bars represent 100 µm in a and c and 20 µm in b and d.
(3.00 MB TIF)

Figure S4 PLP immunization results in limited T-cell occurrence at the hippocampus of APP Tg mice. APP/IFN-γ Tg mice aged 9 months were immunized with PLP and killed 19 days later. Brain sections were immunolabeled for Aβ plaques co-localized with lymphocyte subpopulations and activated microglia as described for Fig. 1. The grayscale images with their corresponding histograms are shown here at 10× (a, c, and e; bars represent 200 µm) and 60× (b, d, and f; bars represent 20 µm).
(3.00 MB TIF)

Figure S5 PLP immunization of APP/IFN-γ Tg mice promotes a slight decrease in Aβ load. APP/IFN-γ mice aged 10 months were left untreated or were immunized with PLP as described in Methods, and killed after 19 days. Brains were removed, sectioned, and immunolabeled with anti-Aβ antibody (green) and counterstained with TO-PRO 3 (blue), as described in Materials and Methods. Representative images of untreated (A) and PLP-immunized (B) mice are shown. Eight sections from each brain were immunolabeled for Aβ and images were analyzed using the Volocity 3D image analysis software. Columns represent the fluorescent area in each brain section of the two analyzed groups (n = 3; means ± SD; P>0.1, Student's *t*-test). Bars represent 200 µm.
(3.00 MB TIF)

Figure S6 A single subcutaneous injection of CFA fails to induce significant clearance of Aβ in APP/PS1/IFN-γ Tg mice. APP/PS1/IFN-γ Tg mice aged 8–9 months were injected with CFA or left untreated, as described in Materials and Methods. On day 19 after immunization mice were killed and brain sections were immunolabeled with Aβ antibodies. Eight sections representing the entire brain were immunolabeled for Aβ and images were analyzed using the Volocity 3D image analysis software. Columns represent the fluorescent area in each brain section of the two analyzed groups (n = 4; means ± SD; P>0.5, Student's *t* test).
(3.00 MB TIF)

Table S1 Percent reduction of Aβ according to the mediolateral position in the hippocampus (%). APP/IFN-γ Tg mice aged 9 months were immunized with Aβ/CFA with and without co-injection of pertussis toxin (PTX). Aβ in brain sections was quantified by immunohistochemical analysis as described in Methods and Supplemental information, Fig. S3. Percent reduction of Aβwas calculated from the average of the immunolabeled area at each mediolateral position of the immunized mice.
(0.03 MB DOC)
