# Supplementary figures and images for: T Cells Specifically Targeted to Amyloid Plaques Enhance Plaque Clearance in a Mouse Model of Alzheimer's Disease
Source: PLoS One. 2010 May 26;5(5):e10830. doi: 10.1371/journal.pone.0010830 (PMC2877087; doi:10.1371/journal.pone.0010830)

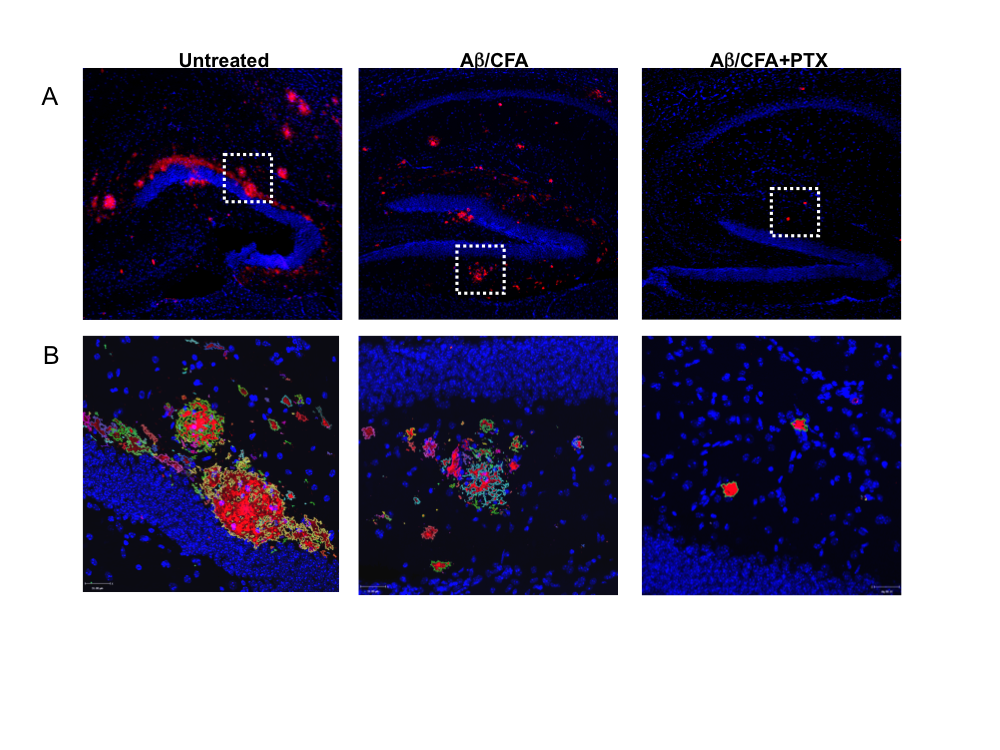

Supplement: Figure S1 — T-cell infiltrates in the parenchyma promote efficient clearance of previously deposited Aβ plaques. APP/IFN-γ Tg mice aged 9 months were immunized with Aβ emulsified in CFA, Aβ emulsified in CFA in conjunction with PTX injections, or left untreated, and examined 35 days later for Aβ in the brain. Brain sections were immunolabeled with anti-Aβ antibody (red) and counterstained with TO-PRO 3 (blue), as described in Materials and Methods. A representative section from each group is shown in A. Higher magnification (×40) of the box in A is shown for each group in panel B. Plaques are marked with colored lines if their fluorescence intensity exceeds the threshold set by the classifier. (3.00 MB TIF) [file pone.0010830.s001.tif]

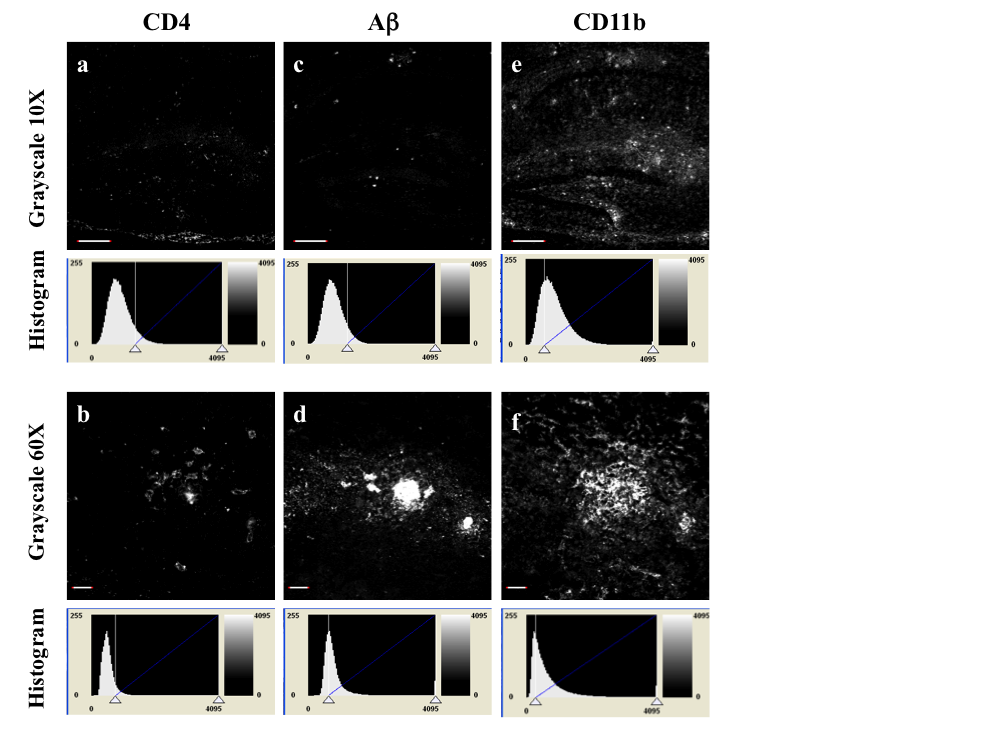

Supplement: Figure S2 — Aβ immunization results in trafficking of T cells to sites of Aβ plaques in the brain parenchyma. APP/IFN-γ Tg mice aged 9 months were immunized with Aβ and killed 19 days later. Brain sections were immunolabeled for Aβ plaques co-localized with lymphocyte subpopulations and activated microglia as described for Fig. 1. The grayscale images with their corresponding histograms are shown here at 10× (a, c, and e; bars represent 200 µm) and 60× (b, d, and f; bars represent 20 µm). (3.00 MB TIF) [file pone.0010830.s002.tif]

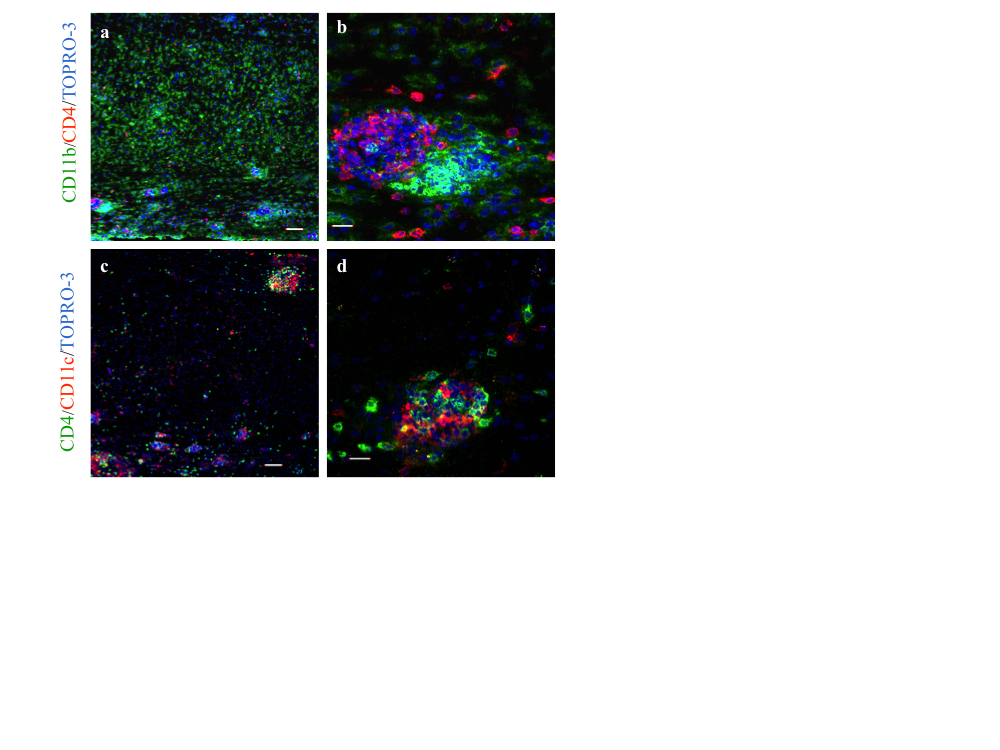

Supplement: Figure S3 — PLP vaccination of APP/IFN-γ Tg mice results in immune-cell infiltration into the spinal cord and cerebellum. APP/IFN-γ Tg mice aged 9 months were vaccinated with PLP/CFA to induce EAE, as described in Materials and Methods, and killed 19 days later. Spinal cord sections were immunolabeled with anti-CD11b antibody (a and b, green), anti-CD11c antibody (c and d, red), and anti-CD4 antibody (red in a and b and green in c and d) and examined under the confocal microscope for inflammatory foci. TO-PRO 3 was used for counterstaining (blue). (b and d) Higher magnifications of inflammatory foci. Bars represent 100 µm in a and c and 20 µm in b and d. (3.00 MB TIF) [file pone.0010830.s003.tif]

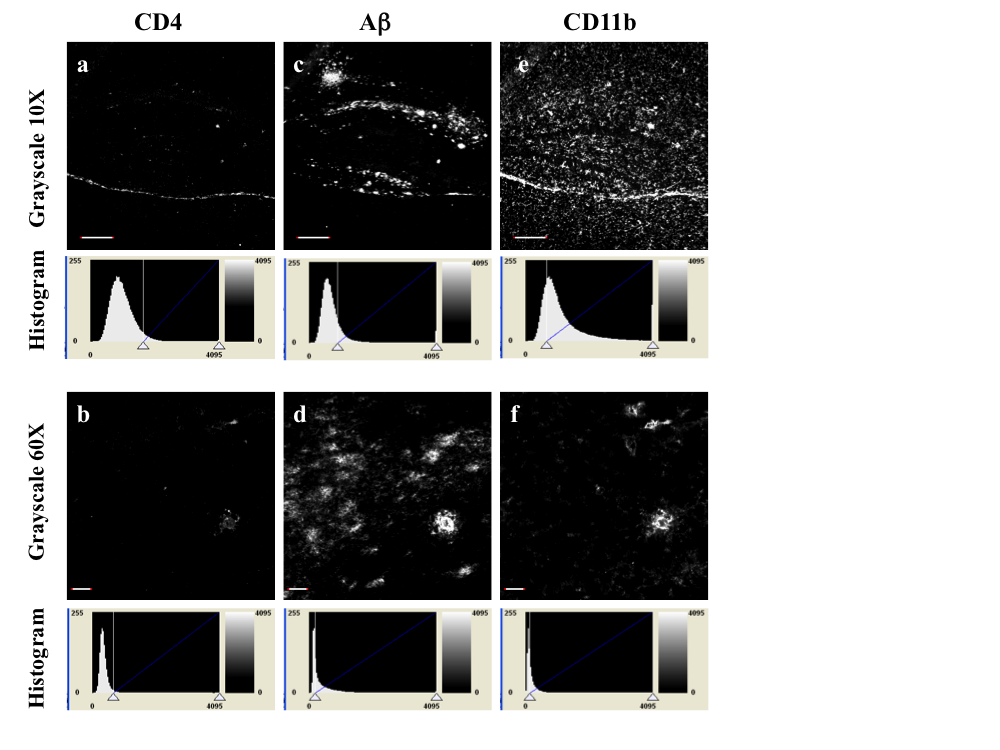

Supplement: Figure S4 — PLP immunization results in limited T-cell occurrence at the hippocampus of APP Tg mice. APP/IFN-γ Tg mice aged 9 months were immunized with PLP and killed 19 days later. Brain sections were immunolabeled for Aβ plaques co-localized with lymphocyte subpopulations and activated microglia as described for Fig. 1. The grayscale images with their corresponding histograms are shown here at 10× (a, c, and e; bars represent 200 µm) and 60× (b, d, and f; bars represent 20 µm). (3.00 MB TIF) [file pone.0010830.s004.tif]

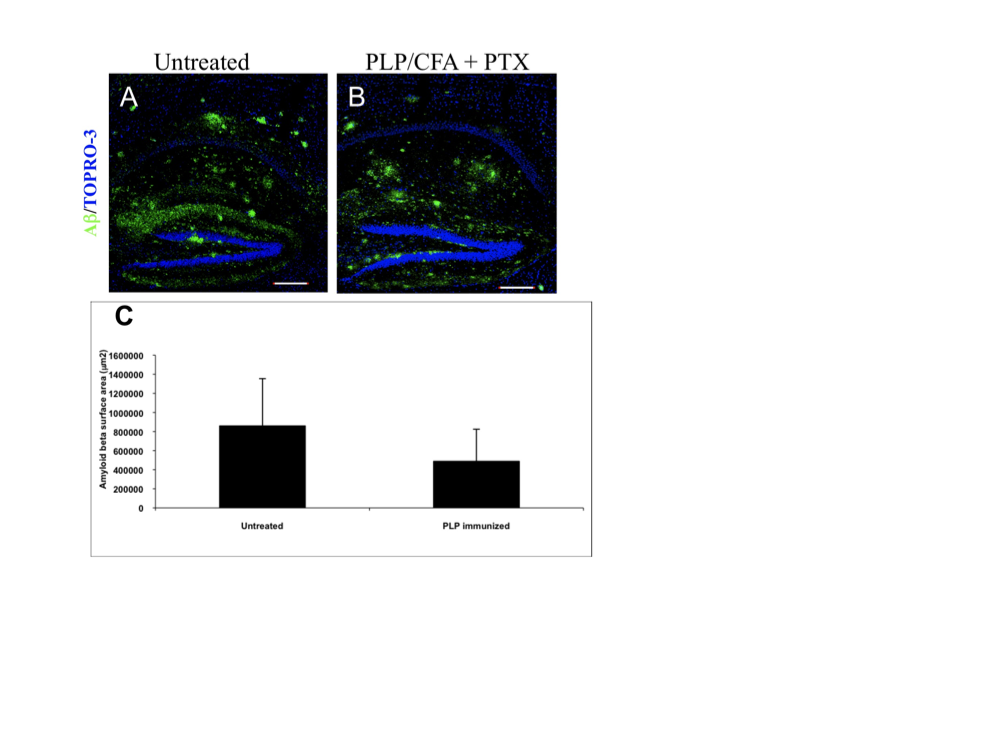

Supplement: Figure S5 — PLP immunization of APP/IFN-γ Tg mice promotes a slight decrease in Aβ load. APP/IFN-γ mice aged 10 months were left untreated or were immunized with PLP as described in Methods, and killed after 19 days. Brains were removed, sectioned, and immunolabeled with anti-Aβ antibody (green) and counterstained with TO-PRO 3 (blue), as described in Materials and Methods. Representative images of untreated (A) and PLP-immunized (B) mice are shown. Eight sections from each brain were immunolabeled for Aβ and images were analyzed using the Volocity 3D image analysis software. Columns represent the fluorescent area in each brain section of the two analyzed groups (n = 3; means ± SD; P>0.1, Student's t-test). Bars represent 200 µm. (3.00 MB TIF) [file pone.0010830.s005.tif]

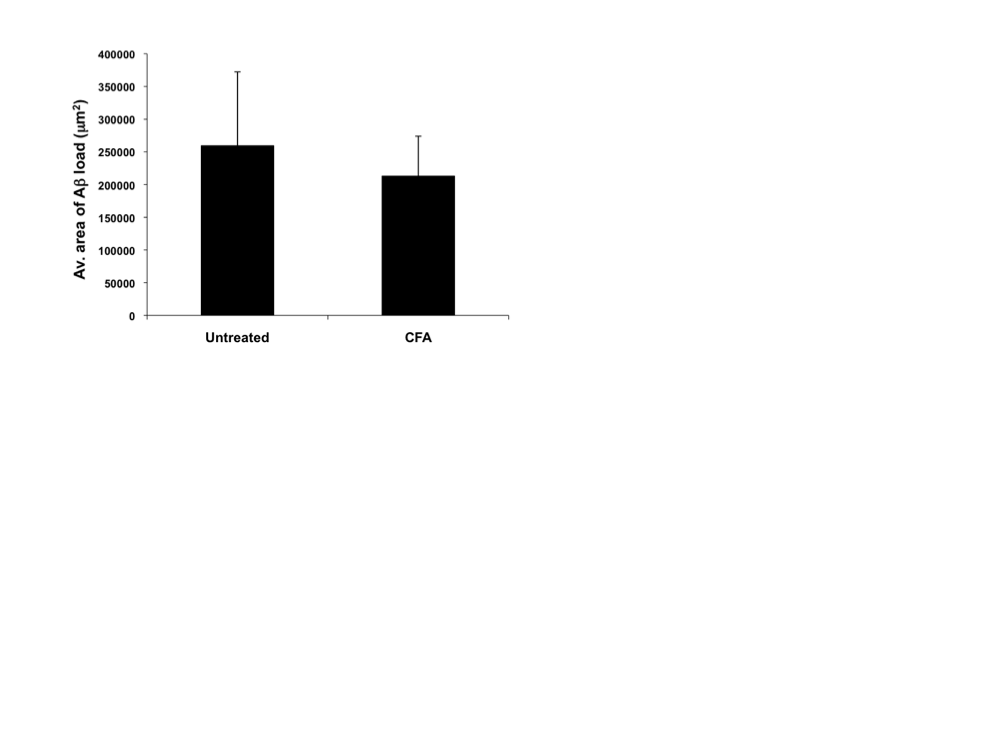

Supplement: Figure S6 — A single subcutaneous injection of CFA fails to induce significant clearance of Aβ in APP/PS1/IFN-γ Tg mice. APP/PS1/IFN-γ Tg mice aged 8–9 months were injected with CFA or left untreated, as described in Materials and Methods. On day 19 after immunization mice were killed and brain sections were immunolabeled with Aβ antibodies. Eight sections representing the entire brain were immunolabeled for Aβ and images were analyzed using the Volocity 3D image analysis software. Columns represent the fluorescent area in each brain section of the two analyzed groups (n = 4; means ± SD; P>0.5, Student's t test). (3.00 MB TIF) [file pone.0010830.s006.tif]
