# Supplementary material for: T Cells Specifically Targeted to Amyloid Plaques Enhance Plaque Clearance in a Mouse Model of Alzheimer's Disease
Source: PLoS One. 2010 May 26;5(5):e10830. doi: 10.1371/journal.pone.0010830 (PMC2877087; doi:10.1371/journal.pone.0010830)
Supplement: Table S1 — Percent reduction of Aβ according to the mediolateral position in the hippocampus (%). APP/IFN-γ Tg mice aged 9 months were immunized with Aβ/CFA with and without co-injection of pertussis toxin (PTX). Aβ in brain sections was quantified by immunohistochemical analysis as described in Methods and Supplemental information, Fig. S3. Percent reduction of Aβwas calculated from the average of the immunolabeled area at each mediolateral position of the immunized mice. (0.03 MB DOC) [file pone.0010830.s007.doc]

| **Slice position**  **(mm)** | **No PTX**  **vs. Cont.** | **PTX**  **vs. Cont.** | **PTX**  **vs. no PTX** |
| --- | --- | --- | --- |
| 0.5–0.9 | 55.01 | 97.35 | 94.11 |
| 0.9–1.35 | 76.54 | 97.20 | 88.08 |
| 1.35–-1.8 | 47.02 | 96.40 | 93.21 |
